# Adaptive Differences in Gene Expression in Farm-Impacted Seedbeds of the Native Blue Mussel *Mytilus chilensis*

**DOI:** 10.3389/fgene.2021.666539

**Published:** 2021-05-20

**Authors:** Marco Yévenes, Gustavo Núñez-Acuña, Cristian Gallardo-Escárate, Gonzalo Gajardo

**Affiliations:** ^1^Programa de Doctorado en Ciencias, Mención Conservación y Manejo de Recursos Naturales, Universidad de Los Lagos, Osorno, Chile; ^2^Laboratorio de Genética, Acuicultura & Biodiversidad, Departamento de Ciencias Biológicas y Biodiversidad, Universidad de Los Lagos, Osorno, Chile; ^3^Laboratorio de Biotecnología y Genómica Acuícola, Centro Interdisciplinario para la Investigación en Acuicultura, Universidad de Concepción, Concepción, Chile

**Keywords:** *Mytilus chilensis*, farm-impacted seedbeds, transcriptome, gene expression, genetic variants, adaptative differences

## Abstract

The study of adaptive population differences is relevant for evolutionary biology, as it evidences the power of selective local forces relative to gene flow in maintaining adaptive phenotypes and their underlying genetic determinants. However, human-mediated hybridization through habitat translocations, a common and recurrent aquaculture practice where hybrids could eventually replace local genotypes, risk populations’ ability to cope with perturbations. The endemic marine mussel *Mytilus chilensis* supports a booming farming industry in the inner sea of Chiloé Island, southern Chile, which entirely relies on artificially collected seeds from natural beds that are translocated to ecologically different fattening centers. A matter of concern is how farm-impacted seedbeds will potentially cope with environmental shifts and anthropogenic perturbations. This study provides the first *de novo* transcriptome of *M. chilensis*; assembled from tissue samples of mantles and gills of individuals collected in ecologically different farm-impacted seedbeds, Cochamó (41°S) and Yaldad (43°S). Both locations and tissue samples differentially expressed transcripts (DETs) in candidate adaptive genes controlling multiple fitness traits, involved with metabolism, genetic and environmental information processing, and cellular processes. From 189,743 consensus contigs assembled: 1,716 (Bonferroni p_*value*_ ≤ 0.05) were DETs detected in different tissues of samples from different locations, 210 of them (fold change ≥ | 100|) in the same tissue of samples from a different location, and 665 (fold change ≥ | 4|) regardless of the tissue in samples from a different location. Site-specific DETs in Cochamó (169) and Yaldad (150) in candidate genes controlling tolerance to temperature and salinity shifts, and biomineralization exhibit a high number of nucleotide genetic variants with regular occurrence (frequency > 99%). This novel *M. chilensis* transcriptome should help assessing and monitoring the impact of translocations in wild and farm-impacted mussel beds in Chiloé Island. At the same time, it would help designing effective managing practices for conservation, and translocation traceability.

## Introduction

The study of adaptive population differences is relevant for evolutionary biology, as it shows how natural selection in heterogeneous environments delineates and maintains adaptive phenotypes and their underlying genetic determinants (Kawecki and Ebert, 2004; Savolainen et al., 2013). Such differences prevail over time if selective local pressures and other evolutionary drivers overcome the homogenizing effect of gene flow (Sanford and Kelly, 2011; Blanquart et al., 2013; Whitlock, 2015). Understanding the genetic basis of adaptive phenotypes helps, eventually, to predict how populations will respond to climate change (Waldvogel et al., 2020) or human-driven habitat translocations; a common aquaculture practice, helpful as a mitigation strategy to increase genetic diversity and reduce extinction risk of inbred and small populations (Šegvić-Bubić et al., 2020). Nevertheless, translocations could increase the risk of loss of locally adapted alleles through the hybridization of divergent populations (Ottenburghs, 2021).

Transcriptomic studies are a valuable tool for genome-wide assessment of expression of coding and regulatory genes, underlying adaptive phenotypes in both model and non-model species (Pespeni et al., 2013; Lockwood et al., 2015; Pastenes et al., 2017; Zhang et al., 2019). They also produce data on nucleotide sequence in expressed genes that may reveal the genetic diversity of transcripts of candidate genes controlling fitness traits (DeBiasse and Kelly, 2016), providing an opportunity to assess gene-level standing variation (Bitter et al., 2019). The transcriptome is considered a phenotype by itself (Schulte, 2004), with a heritable pattern of expression (Gu et al., 2019), which can perceive subtle environmental changes due to its fine-grained nature, often not detected at the organismic level (DeBiasse and Kelly, 2016). It integrates the molecular and functional complexities distributed over the entire genome with higher organization levels such as fitness-related traits (Lockwood et al., 2015).

The endemic Chilean blue mussel *Mytilus chilensis*, a close relative of the *M. edulis* species complex of the northern hemisphere (Larraín et al., 2018), is a good model to address problems in ecology (Curelovich et al., 2016), ecophysiology (Duarte et al., 2018), adaptation and evolution (Araneda et al., 2016; Gaitán-Espitia et al., 2016). It is a keystone taxon in the ecosystem regulating phytoplankton, nutrient flow and contributes to remineralizing organic deposits in the sediment (Gallardi, 2014). It inhabits rocky substrates in the intertidal and subtidal zones along the southern Pacific Ocean, from latitude 38°S (Bío-Bío Region) to 53°S (Magellan Straits) (Molinet et al., 2015; Oyarzún et al., 2016; Larraín et al., 2018; Jahnsen-Guzmán et al., 2021). As a gonochoric species, with an annual gametogenic cycle, sexual maturity occurs in spring-summer (Oyarzún et al., 2011), then fertilization and development of the planktonic larvae take place. Since larvae can drift in the water column between 20 and 45 days before settlement (Toro et al., 2004; Ruiz et al., 2008), it has an estimated dispersal potential of up to 30 km (Barría et al., 2012), allowing different degrees of gene flow among populations within that distance.

The species boosts a booming farming industry, concentrated in the inner sea of Chiloé Island (41–44°S), an area full of fjords and protected bays with high phytoplankton productivity. However, it exhibits a highly inter-annual environmental variability and a marked north-south difference in temperature, salinity, ocean current circulation, and concentration of chlorophyll-a (Castillo et al., 2015; Martínez et al., 2015; Lara et al., 2016). This industry depends entirely on seed collection from natural beds (Astorga et al., 2020), which are threatened by ocean warming and increasing acidification, affecting the mussels’ fitness through the biomineralization process of shell growth, reproductive performance and recruitment (Castillo et al., 2017; Díaz et al., 2018; Malachowicz and Wenne, 2019; Mlouka et al., 2019). Likewise, the highly extractive pressure of selected phenotypes and translocations from seedbeds to fattening centers, a practice with poor traceability, hybridizes divergent populations eroding genetic diversity and affecting the fitness landscape (Ottenburghs, 2021). Given the importance of genetic diversity for evolutionary change and adaptation to unpredictable environments (Hoban et al., 2020; Laikre et al., 2020), there is a need to investigate adaptive differences in natural seedbeds impacted by the industry (henceforth farm-impacted seedbeds). Nevertheless, the literature on intraspecific genetic diversity and adaptive population differences of *M. chilensis* is scarce, making it difficult to anticipate how the species could respond to environmental perturbations, habitat translocations, and heavy exploitation.

Studies with neutral nuclear markers (microsatellites) report low genetic differentiation (F_*ST*_ = 0.042) among wild mussel’s samples distributed along a latitudinal gradient of temperature, salinity, and oxygen availability; including some farm-impacting seedbeds (Larraín et al., 2012, 2015; Araneda et al., 2016; Astorga et al., 2018, 2020). The use of adaptive Single Nucleotide Polymorphic markers (outlier SNPs in the DNA), obtained by RAD-Seq suggests that mussel populations may retain local adaptations (Araneda et al., 2016). Previous studies have explored in transcriptomic differences with a selected number of candidate genes in which natural populations are compared along a latitudinal gradient (39–43°S) (Núñez-Acuña et al., 2012). Results show that mussels experiencing high temperatures up-regulated genes coding for chaperones Hsp70 and Hsp90, known to protect against environmental stress, while individuals exposed to low salinity up-regulated gene coding for Mytilin B involved in immunological defense. Similarly, the expression of SOD-CuZn and Catalase genes regulating oxidative stress, appeared related to the local concentration of chlorophyll-a. Two follow-up experimental transcriptomic studies exposed mussels to saxitoxin, one of the main phycotoxins, causing paralytic shellfish poisoning. One of them identified 13 differentially expressed candidate genes, related to cellular stress and immune response (Núñez-Acuña et al., 2013), while the other showed strong up-regulation of genes encoding for the Recognition Receptor Proteins (RRP family), also involved in immune response (Detree et al., 2016). About 20,306 polymorphic genetic variants were detected in transcripts of genes of the immune system, some of which responded to other marine toxins (Núñez-Acuña and Gallardo-Escárate, 2013). A reciprocal transplant experiment (Osores et al., 2017) evaluated the reaction norms of morphological, biochemical, physiological, and life-history traits between two contrasting local environments; estuarine (39°S) vs. coastal (41°S), finding no significant differences at the organismic level. However, the gene encoding for the chaperone Hsp70 showed a differential expression in both, locals and transplanted individuals from both locations. A recent transplant experiment (Jahnsen-Guzmán et al., 2021) demonstrated that *M. chilensis* individuals are adapted to the subtidal environment (4 m depth), as they exhibit significantly higher fitness (growth and calcification rates) than those transferred to the intertidal environment (1 m depth), which showed increased metabolic stress. These examples demonstrate the extreme variability of the mussel environment and their ability to cope with perturbations, be it habitat translocations or environmental oscillations, with plastic and adaptive response. All these studies suggest the need to discover genome-wide marker genes and underlying fitness traits to get insight into how perturbations affect the adaptative landscape (Hoban et al., 2020; Laikre et al., 2020).

A genome-wide transcriptomic study offers a more realistic and environmentally sensitive approach (Lockwood et al., 2015; DeBiasse and Kelly, 2016) to eventually understand how *Mytilus chilensis* could respond to climatic and human-driven perturbations such as translocations. Likewise, it should provide genomic resources for conservation and exploitation traceability to design effective management practices. The goals of this study are (1) to assemble the *de novo* transcriptome of *M. chilensis* from two ecologically contrasting farm-impacted seedbeds supporting the industry; (2) to test tissue-specific (gill and mantle) differences in expressed transcripts of candidate genes and site-specific (between seedbeds) differences in transcriptomic genetic variants of candidate adaptive genes; (3) and to provide novel genome resources to investigate adaptive differences in wild and farm-impacted mussels, to avoid population loss in a scenario of multiple environmental perturbations, including heavy aquaculture exploitation of natural seedbeds.

## Materials and Methods

### Study Sites and Sampling

*Mytilus chilensis* individuals were sampled from two places located in the main seedbeds supporting the industry (Larraín et al., 2012), approximately 250 km distant: Cochamó (41°28′23.77′ ‘S– 72°18’38.61″W), located in the Reloncaví fjord, north of Chiloé Island, an estuary with continuous input of freshwater and vertical stratification of water characteristics. The other site was Yaldad (43°07′14.63′ ‘S– 73°44’25.72″W), in the southern part, a bay exposed to open sea influence. Seawater parameters of temperature (°C), currents (m/s), salinity (psu), and age of seawater (days) as an estimate of dissolved oxygen content (Pinilla et al., 2020), were obtained for both locations between June 2017 and May 2018 (0 to 10 m of depth), period overlapping with sampling dates. The environmental raw data were collected from the CHONOS database^1^, managed by the Instituto de Fomento Pesquero, IFOP (Institute of Fisheries Enhancement), for both locations. The software ODV v5.3^2^ allowed the visualization and displaying of data.

Size and growth rate differences between adult mussels from both locations were compared at two different dates, one on April 26 of 2018 and 91 days later, on July 28. Samples were taken from the same hanging collectors, distant approximately 200 m from the shore and between 4 to 10 m of depth. According to the local farmers, sampled individuals corresponded to larvae settled in submerged hanging collectors and kept for almost 3 years in the same place. One hundred healthy (assessed by siphoning activity) adult mussels were initially sampled, and the same quantity on the second sampling. From the 100 individuals sampled on July 28 (second sampling), 15 were randomly taken from each location to perform the taxonomic affiliation analysis. Other 15 individuals from the second sampling were left to construct the cDNA libraries to be sequenced.

Permits were not required to collect *M. chilensis*, because they are unregulated, and the collection did not involve endangered or protected species in the study locations. The dissection of animals and tissue processing followed the protocols established by Universidad de Los Lagos and Universidad de Concepción. Mussels taken from the hanging collectors were washed with local seawater, stored in separate sterile bags in a cooler at 10 ± 2°C, and transported to the laboratory. Individual length (cm), width (cm), and shell thickness measurements were registered with a Vernier caliper. As a proxy of the mean size, the relationship between weight and length was estimated by the “power transform” function available in R, v4.0.3. Normalized weight values (λ = 0.295) and ANOVA, allowed comparing mean sizes between locations. Moreover, the obtained mean size values were compared in order to estimate growth rate values by location between the first (day 0, April 26) and second sampling (day 91, July 28). The Bonferroni and Tukey were considered as *a posteriori* tests to assess growth differences after 91 days.

Fifteen individuals from the second sampling date (on July 28) at each location were randomly taken to perform total DNA extraction for taxonomic affiliation analysis, and the same number of mussels were considered for RNA-Seq. Samples from gill tissue were dissected and collected in 1 mL ethanol 70% (v/v) and stored at −20°C within four h after collection. Contrarily, samples from gills and mantle tissues were dissected and collected in 1 mL of EZNA RNA lock reagent (OMEGA BioTek^TM^), and stored at −80°C within 4 h after collection.

### Taxonomic Affiliation

The DNA extraction was performed from the collected gill tissues, using the EZNA Tissue DNA Kit (OMEGA BioTek^TM^) and following the manufacturer’s instructions. The taxonomic affiliation was carried out using two molecular RFLP assays for the mitochondrial COI-*Xba*I (Fernández-Tajes et al., 2011), and the nuclear Me15/Me16-*Aci*I (Larraín et al., 2012). The COI-*Xba*I L and R primers were used with a conventional PCR to obtain a 233 bp amplicon, with a restriction site only in M. chilensis, but not in the non-native species *M. edulis* and *M. galloprovincialis*. The nuclear Me15/Me 16-*Aci*I marker corresponds to codominant nuclear gene Glu, which encodes a segment of one of the sticky mussel foot byssus proteins. Using the M15/Me16 L and R primers, an amplicon of 180 bp for *M. edulis*, and another of 126 bp for *M. galloprovincialis* and *M. chilensis* were obtained. The restriction enzyme *Aci*I cut these fragments only in *M. edulis* and *M. galloprovincialis*, not *M. chilensis*. The analysis of these two molecular RFLP test results indicated, with reasonable certainty, that the sampled individuals who participated in this study corresponded to Mytilus chilensis. These results are in Supplementary Figure 1.

### RNA Extraction, cDNA Library and Sequencing

High-quality total RNA was individually isolated from gills and mantle tissues of individuals from the last sampling using TRIZOL (Invitrogen^TM^), following manufacturer instructions. RNA integrity was visualized with electrophoresis in 1.2% MOPS/formaldehyde agarose gels stained with 0.01% GelRed (Biotium^TM^) using TapeStation 2200 (Agilent Technologies^TM^) with the R6K reagent kit. Purity and concentration were checked by spectrophotometry (NanoDrop Technologies) and fluorescence (Qubit 4, Thermo Scientific^TM^). Some of these results are in Supplementary Figure 2. RNA extracts with 260/280 and 260/230 ratio >2.0 and RNA Integral Number (RIN) estimation > 9, were selected for cDNA library construction.

Six cDNA libraries per location were constructed, three for each tissue (replicates). Each library contained equal quantities of total RNA from 5 randomly selected individual extractions. These mixed RNAs were precipitated overnight, in 2 volumes of absolute ethanol and a 0.1 volume of 0.3 M sodium acetate at −80°C. Thus, a total of 12 high-quality libraries were constructed using TrueSeq Stranded mRNA LT Sample Prep Kit and protocol (Illumina Platformc^TM^), and whole RNA-Seq sequenced in an Illumina HiSeq 4000 Platformc^TM^ with a 100 paired-end approach. The data presented in this study are deposited in the GenBank repository, under the Bio Project accession number PRJNA630273 (Supplementary Table 1).

### The *de novo* Transcriptome Assembly

Trimming of raw data for each library and *de novo* assembly was done with CLC Genomic Workbench software v21.0.3 (Quiagen Bioinformaticsc^TM^) using restrictive filters to obtain clean reads (quality score of 0.05, remotion of low-quality sequences, mismatch cost of 2 and 3 for insertions and deletions, length of 0.8, and similarity fractions of 0.9 with a maximum number of hits for a read of 10). For the reference library based on all samples, regions with low coverage (threshold of 20) were removed. After that, the resulting gene library for the whole transcriptome contains 189,743 consensus contigs with a minimal length of 200 bp. This reference gene library was used for mapping the clean reads and for the differential expression analyzes.

### RNA Seq and Differential Expression Data

Matching reads for all RNA Seq samples were sorted out to generate a differential expression dataset, using as referent the 189,743 consensus contigs (reference gene library) derived from the *de novo* assembly. Different statistical filters were also used to avoid confirmation biases and false positives in selecting differentially expressed transcripts (DETs) during the comparative process. The normalization and quantification of the samples’ clean reads was automatically performed by the CLC software, using the Trimmed Mean of M values method and following the EdgeR approach. The number of transcripts per million (TPM) represented a proxy of gene expression measurement to detect DETs. It was estimated as a global alignment with the reference gene library, with a mismatch cost of 2 and 3 for insertions and deletions, length of 0.8, and similarity fractions of 0.8, with 10 maximum number of hits as an additional filter. After that, a principal component analysis (PCA) by replicate was performed to identifying outlying samples and provided a general perspective of the variation in the reads counts for each transcript in the dataset. The transcripts with zero reads count or invalid values were removed.

The differential expression analysis considered a negative binomial generalized linear model (GLM) and the Wald test to determine if differences due to sampling origin (controlled by replicate and tissue) were different from zero. To correct the differences in library size between samples and the replicates effect, fold changes (FC) were estimated from the GLM. Using Euclidean distances, FC ≥ | 4|, False Discovery Rate (FDR) corrected p_*value*_ ≤ 0.05, and average linkage between clusters, this dataset grouped by tissue and location was visualized in a clustering heat map. After that, the samples were compared as follows: (i) intra- location by tissue, i.e., samples of different tissues from individuals of the same location, (ii) inter- location by tissue, samples of the same tissue of individuals from different locations, and (iii) by location, samples from different locations regardless of the tissue. For that, restrictive filters were also used, an FC ≥ | 100| and Bonferroni corrected p_*value*_ ≤ 0.05 for intra- and inter- location by tissue comparisons and FC ≥ | 4| and Bonferroni p_*value*_ ≤ 0.05 for comparison by location. Those contigs who passed these filters were recognized as DETs. After that, DETs were extracted and annotated.

### DETs Annotations and Functional Categorization

Contigs screened as differentially expressed transcripts (DETs), by intra- and inter-location by tissue and by location comparisons were annotated using the BLASTx tool of the CLC software (e_*value*_ ≤ 1E-05) and the UniprotKB/SwissProt databases. For the description of putative transcripts, homology searches considered the NCBI EST database using the tBLASTx algorithm. For their functional characteristics, DETs sequences were gene-enriched using a hypergeometric distribution model performed in the KOBAS online server (Xie et al., 2011) and the related mollusk *Crassostrea gigas* as referent. The sequences were functionally categorized using the Kyoto Encyclopedia of Genes and Genomes pathways database (KEGG terms) and the Fisher exact test (p_*value*_ ≤ 0.05) as enrichment test for over-represented KEGG terms. For visualization, the enrichment ratio was estimated by dividing the input number (number of DETs matched with KEGG ID terms) and the background input number (total of genes by category of the KEGG database).

### Genetic Variants

The Variant Detection tool of CLC software was used to detect genetic variants (GVs) in the sequences of the transcriptome of samples from both locations, namely single nucleotide variants (SNV), multiple nucleotide variants (MNV), deletions, insertions, and replacements. Two new assemblies were obtained (one per location regardless of the tissue), and overlap settled for a minimum contigs length of 200 bp, a mismatch cost of 2, linear gap cost for insertions and deletions, a length fraction of 0.8 and 0.9 of similarity. The mapping was performed over the reference gene library, and previously selected differential expressed transcripts (DETs), i.e., reads by location, were mapped back over 189,743 consensus contigs and 7,900 DETs selected from the differential expression analysis. For this, a strict overlap for reads and coverage filters was used. Non-specific matches and duplicated reads were ignored, and a minimum coverage = 10, minimum count = 2, and minimum frequency nucleotide variant = 5% were considered. Noise and quality filters were set in a neighborhood radius of 5 nucleotides, minimum central quality of 20 with minimum neighborhood quality of 15. False positives, which are variants whose read position distribution was significantly different from the reference gene library, and DETs at false discovery rate (FDR) corrected p_*value*_ < 0.01, were removed. Those GVs showing regular occurrence (frequency > 0.99) in each mapping by location were extracted and annotated since they likely represent differentially expressed monomorphic SNPs in the DNA, segregating in the overall population and yet fixed between samples of the two locations analyzed.

## Results

### Environmental Conditions

**FIGURE 1:**
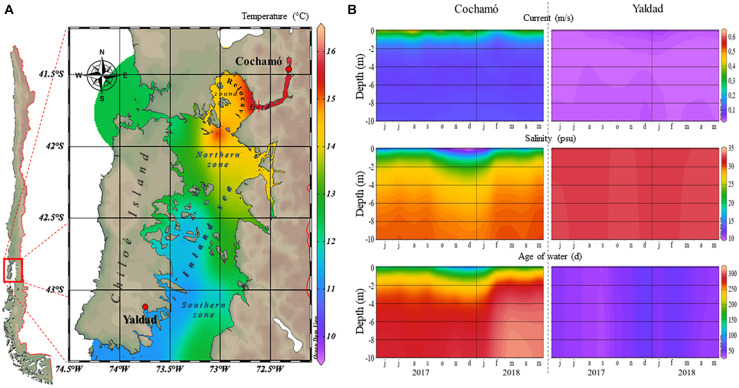
**(A)** A map indicating the geographical locations of the samplings, Cochamó (north) and Yaldad (south), and seawater temperature for March 2018. **(B)** Seawater conditions variations between June 2017 and May 2018.

The analysis of 1-year data of water conditions (from June 2017 to May 2018) collected from CHONOS, revealed large differences in some oceanographic conditions of both sampling locations (Figure 1). Cochamó (0 to 10 m of depth) exhibited vertical stratification and higher temperatures, higher marine currents, and higher water age (retention time) than Yaldad, but lower salinity. These observations support the idea that there are two oceanographically different zones in the inner sea of Chiloé Island, the northern area where Cochamó is located, and the south where Yaldad is. During the first sampling (April 2018), the seawater conditions for Cochamó were: temperature = 12.2°C and salinity = 19 psu; whereas Yaldad showed 11.6°C and 31.4 psu. After 91 days (July 2018), Cochamó registered 10.4°C and 20.5 psu; whereas Yaldad 9.6°C and 32 psu.

### Size Comparison

Mean size comparisons between Cochamó and Yaldad samples, estimated as the length-corrected weight (λ = 0.295), were significantly different (p_*value*_ < 0.05). Thus, Cochamó individuals were smaller (mean size: 3.79 ± 0.63 g) at the first sampling than in Yaldad (5.75 ± 0.67 g). At the second sampling, Cochamó individuals exhibited a slightly smaller size (5.19 ± 0.39 g) than those of Yaldad (6.14 ± 0.55). The estimate of the growth rate for 91 days, i.e., from the first sampling day (April 26, 2018) to the second (July 28, 2018), for Cochamó individuals was higher (0.015 g/day) than Yaldad (0.004 g/day).

### Differential Expression Analysis and Annotations

**FIGURE 2:**
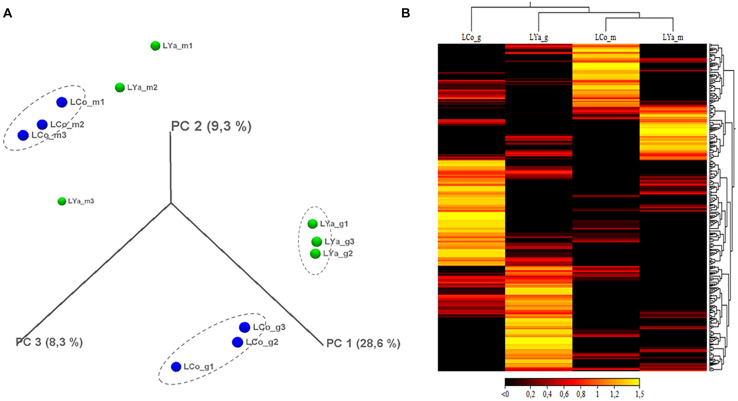
3D-PCA plot **(A)** resulting from the principal components analysis by replicating local samples from Cochamó and Yaldad. Heat map **(B)** representing a visual distribution by tissue and location of the up-regulated DETs of individuals from Cochamó and Yaldad. LCo, local individuals from Cochamó and LYa, locals from Yaldad. _m, mantle samples and _g, gill samples.

The *Mytilus chilensis* transcriptome totalized 89.63 Giga bases (Gb) of sequences distributed in 890,600,608 trimmed raw reads (Table 1). The *de novo* assembly yielded a reference gene library of 189,743 consensus contigs, between 201 and 16,311 bases (b) long, with an average of 532 b and 100.91 Mb in total. Different reads count by transcript and differentially expressed transcripts (DETs) were detected for each intra- and inter-location by tissue and by location comparison from the reference gene library mapping. The results from PCA analysis (Figure 2A) showed that the differences in reads count by transcript between replicates for the same tissue are smaller than the differences between replicates for different tissues and locations. It allowed to recognize and differentiate samples tissues from gills and mantle of individuals from both locations. On the other hand, the grouped replicate by tissue and location showed differences in the expression profile of each one; however, samples from mantle tissue of individuals from Cochamó and Yaldad were more similar between them than samples from gills tissue as is showed in the heatmap of Figure 2B. It indicated considerable differences between sampled tissues.

**TABLE 1:** Summary of the Illumina sequencing characteristics from the Chilean mussel *Mytilus chilensis* transcriptome inferred from native individuals habiting two seedbeds, Cochamó and Yaldad.

Descriptor	*de novo* assembly	Reference gene library	Cochamó	Yaldad
Number of Reads (M)	890.60		449.18	441.80
Average length (b)	101		101	101
Matched (M)	665.02		340.19	331.22
Number of Contigs	463,359	189,743	339,916	327,650
Average (b)	506	532	524	534
Minimum	101	201	79	80
Maximum	23,367	16,311	26,019	22,840
Total bases (M)	234.67	100.91	178.06	175.03

*M, mega; b, bases.*

#### Intra-Location by Tissue Comparison

**FIGURE 3:**
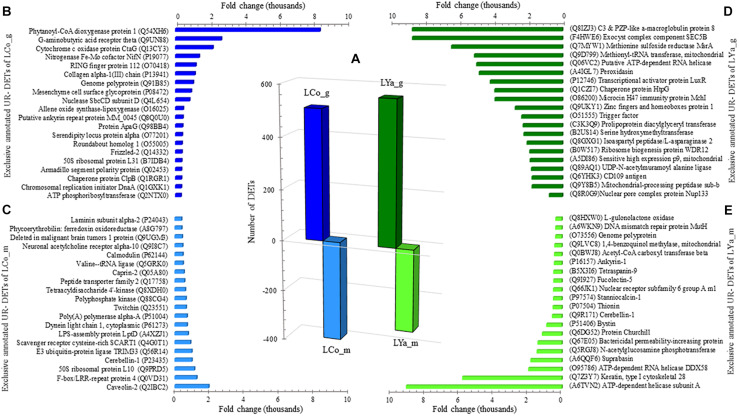
Intra-location by tissue comparison. The number (bars) of differentially expressed transcripts (DETs) by location are in the central plot **(A)**; the number of up-regulated (UR-) DET from mantle tissue by location is in negative values. Also are showed the top twenty exclusive annotated UR-DETs from gills **(B)** and mantle **(C)** tissues for samples from Cochamó (left) and Yaldad (right, **D,E**, respectively). LCo, local individuals from Cochamó and LYa, locals from Yaldad. _m, mantle samples and _g, gill samples.

The transcript per million (TPM) values between tissue estimated the number of DETs for both locations (Supplementary Table 2). The number of gills DETs was higher than those of the mantle. Thus, 508 out 833 DETs appear up-regulated in gill samples of individuals from Cochamó (LCo_g), while the mantle (LCo_m) presented 391 DETs (Figure 3A). Contrarily, samples from Yaldad showed a total of 883 DETs, 560 of them up-regulated in gills (LYa_g) and 323 in the mantle (LYa_m).

The search for DETs similarities at the UniprotKB/SwissProt database produced significant blast matches to different annotated genes. For Cochamó, the available BLAST hit of up-regulated (UR-) DETs of LCo_g was 411 and 354 UR- DETs for LCo_m. The most relevant glossed match for LCo_g (FC = 38,300) was for the Insoluble Matrix Shell Protein 6 (IMSP6, GenBank accession P86987), a novel protein of the acid-insoluble organic matrix of the shell calcification processes (Supplementary Table 3). The most relevant for LCo_m (FC = 17,554) was Caveolin-3 (CAV3, GenBank accession P51638), a protein related to other scaffolding protein within caveolar membranes, which can interact with G-protein and potassium channels. Since that, some annotations found for these DETs were shared by both tissues; to get more information about differences in gene expression between tissues, those exclusive annotated UR- DETs by tissue were recognized. Sorted by FC, the top twenty exclusive annotated UR- DETs of LCo_g, are in Figure 3B, where the Phytanoyl-CoA dioxygenase protein 1 (PHYD1, GenBank accession Q54XH6), protein with dioxygenase and oxidoreductase activity, is highlighted with a FC = 8,431. The top twenty exclusive annotated UR- DETs of LCo_m, are in Figure 3C, highlighting the Caveolin-2 (CAV2, GenBank accession Q2IBC2) with a FC = 1,959. Concerning Yaldad, the available BLAST hit of UR- DETs of LYa_g was 479 and 291 UR- DETs for LYa_m. The most relevant annotated match for LYa_g (FC = 38,655) was the Peroxidasin homolog (PXDN, GenBank accession Q3UQ28), related to peroxidative reactions and in the formation of extracellular matrix (Supplementary Table 3). The most relevant for LYa_m (FC = 26,455) was Gastrokine-2 (GKN2, GenBank accession Q9CQS6), the protein participating in the formation of heterodimer with the gastric tumor suppressor peptide TFF1. Exclusive annotated UR- DETs by tissue were also recognized in these samples. Sorted by FC, the top twenty exclusive annotated UR- DETs of LYa_g, are in Figure 3D, with the peripheral membrane protein being relevant with FC = 8,738. It interacts with heparin C3 and PZP-like alpha-2-macroglobulin domain-containing protein 8 (CPMD8, GenBank accession Q8IZJ3). The top twenty exclusive annotated UR- DETs of LYa_m, are in Figure 3E, highlighting (FC = 1,959) the ATP-dependent helicase subunit A (ADDA, GenBank accession A6TVN2), a DNA- binding protein related to DNA damage and repair.

#### Inter-Location by Tissue Comparison

**FIGURE 4:**
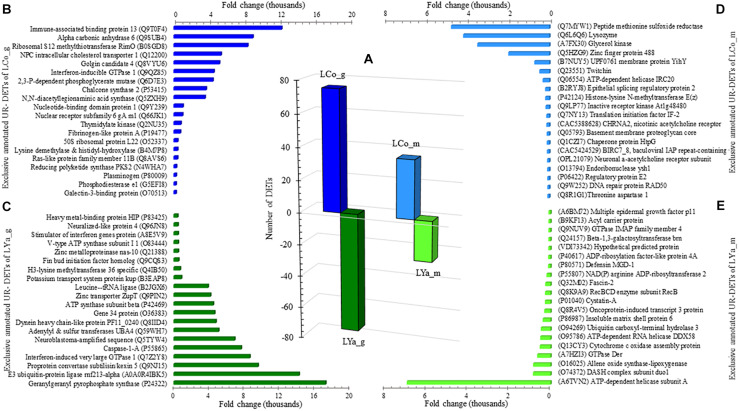
Inter-location by tissue comparison. The number (bars) of differentially expressed transcripts (DETs) by tissue are in the central plot **(A)**; the number of up-regulated (UR-) DETs from gills and mantle tissues for Yaldad are in negative values. Also are showed the top twenty exclusive annotated UR-DETs from gill samples from Cochamó **(B)** and Yaldad **(C)**. Top twenty exclusive annotated UR-DETs from the mantle are in **(D)** for Cochamó and **(E)** samples for Yaldad. LCo, local individuals from Cochamó and LYa, locals from Yaldad. _m, mantle samples and _g, gill samples.

The number of DETs by tissue in both locations was determined (Supplementary Table 4) and the results indicated that the number of gills DETs was higher than those of mantle. Thus, for the gill samples, 75 out 149 were UR- DETs of LCo_g and 74 of LYa_g (Figure 4A). For the mantle samples, 36 out 61 were UR- DETs of LCo_m, and 25 UR- DETs of LYa_m.

The available BLAST hit of UR- DETs of LCo_g was 60 and 66 for LYa_g (Supplementary Table 5). The most relevant annotated match (FC = 12,231), and also exclusive, was the Immune-associated nucleotide-binding protein 13 (IAN13, Genbank accession Q9T0F4), a protein that belongs to the TRAFAC class (GTPase family) related to growth regulator factors (Figure 4B). The most relevant and exclusive UR- DET for LYa_g (FC = 17,217), was the Geranylgeranyl pyrophosphate synthase (GGPPS, GenBank accession P24322), involved in the isoprenoid biosynthesis (Figure 4C). Concerning mantle, the available BLAST hit of UR- DETs of LCo_m was 28 and 22 for LYa_m. The most relevant annotated for LCo_m (FC = 4,738) was the Peptide methionine sulfoxide reductase (MSRA, GenBank accession Q7MYW1), which has a role in repairing inactivated-by-oxidation proteins (Figure 4D). The most relevant and exclusive UR- DET for LYa_m (FC = 6,902) was the ATP-dependent helicase/nuclease subunit A (Figure 4E).

#### Comparison by Location

**FIGURE 5:**
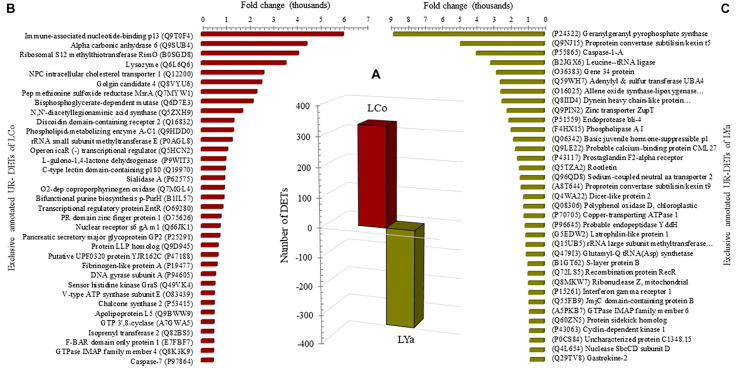
Comparison by location. The number of differentially expressed transcripts (DETs) by location (bars) are in the central plot **(A)**; the number of the up-regulated (UR-) DETs of samples from Yaldad are in negative values. Also are showed, sorted by fold change, the top thirty-five exclusive annotated UR-DETs for samples from Cochamó **(B)** and Yaldad **(C)**. LCo, local individuals from Cochamó and LYa, locals from Yaldad.

Regardless of the tissue, the number of DETs for each location was determined (Supplementary Table 6) with Cochamó individuals (LCo) showing 334 UR- DETs and Yaldad (LYa) 331 (Figure 5A). The search for DETs similarities at the UniprotKB/SwissProt database also produced significant blast matches to different annotated genes. The available BLAST hit of up-regulated UR- DETs was 285 for both UR- DETs of LCo and LYa (Supplementary Table 7). The most relevant annotated match for LCo (FC = 6,011), was the Immune-associated nucleotide-binding protein 13, the same UR- DET was observed as exclusive UR-DET in samples of LCo_g from the inter-location by tissue comparison (Figure 5B). This also was observed in samples of LYa, the most relevant annotated UR- DET (FC = 8,760) was the Geranylgeranyl pyrophosphate synthase, also observed as exclusive UR-DET in LYa_g from the inter-location by tissue comparison (Figure 5C).

### KEGG Categorization of DETs

The enrichment KEGG terms for the DETs sequences selected, derived from the differential expression analysis (after removing those redundant), showed a diversity of functions greater for gill than the mantle. It also showed that the main differences in function involved metabolism, genetic and environmental information processing, and cellular processes.

#### Intra-location by Tissue KEGG Terms

**FIGURE 6:**
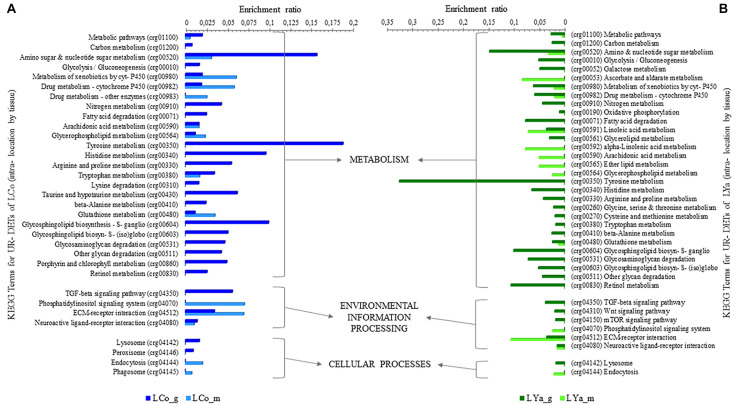
KEGG categorization of differentially expressed transcripts (DETs) for intra-location by tissue comparison. The KEGG terms represented by up-regulated (UR-) DETs from Cochamó are in **(A)** and those represented by samples from UR-DETs from Yaldad in **(B)**. LCo, local individuals from Cochamó and LYa, locals from Yaldad. _m, mantle samples and _g, gill samples.

The intra-location KEGG terms for tissue comparisons showed that 29 out 33 KEGG terms were represented by 97 UR-DETs of Cochamó gill samples (LCo_g), and 14 by 48 UR- DETs of mantle samples (LCo_m). Contrarily, 31 out 38 KEGG terms by 127 UR- DETs of Yaldad gill samples (LYa_g) and 15 by 42 UR- DETs of Yaldad mantle samples (LYa_m) (Supplementary Table 8). Figure 6 shows the differences, expressed as enrichment ratio (input number/background input number), between the KEGG terms represented by UR- DETs resulting from this comparison. The representative KEGG terms appear involved with metabolism, particularly carbohydrates, xenobiotics, energy, lipids, amino acids, glycan, cofactors and vitamins. Environmental information processing through signal transduction and signaling molecules interaction and cellular processes related to transport and catabolism. The most relevant KEGG terms, represented by the samples of LCo_g, were related to amino sugar and nucleotide sugar metabolism (crg00520), tyrosine (crg00350), histidine (crg00340), taurine and hypotaurine (crg00430), and glycosphingolipid biosynthesis-ganglio series (crg00604). KEEG terms involved with environmental information processing were related to TGF-beta signaling pathway (crg04350), and cellular processes linked to transport and catabolism such as lysosome (crg04142), and peroxisome (crg04146) (Figure 6A). On the contrary, the most relevant KEGG terms correspond to LCo_m and are involved with the metabolism of xenobiotics (crg00980) and drugs (crg00982) through cytochrome P450, and glutathione (crg00480). KEEG terms involved with environmental information processing were related to phosphatidylinositol signaling system (crg04070) and ECM-receptor interaction (crg04512), and those involved with cellular processes were related to endocytosis (crg04144), and phagosome (crg04145). Concerning Yaldad, the most relevant KEGG terms, represented by the samples of LYa_g, were those related to metabolism of amino sugar and nucleotide sugar metabolism (crg00520), xenobiotics by cytochrome P450 (crg00980), fatty acid degradation (crg00071), tyrosine (crg00350), and retinol (crg00830). KEEG terms involved with environmental information processing were related to TGF-beta (crg04350), Wnt (crg04310), and mTOR signaling pathways (crg04150), and ECM-receptor interaction (crg04512). Those KEGG terms involved with cellular processes were related to lysosome (crg04142) (Figure 6B). On the other hand, the most relevant KEGG terms represented by samples of LYa_m involved with metabolism were those related to ascorbate and aldarate (crg00053), linoleic acid (crg00591), arachidonic acid (crg00590), and ether lipid (crg00565). KEEG terms involved with environmental information processing were related to ECM-receptor interaction (crg04512), and those involved with cellular processes were related to endocytosis (crg04144).

#### Inter-Location by Tissue KEGG Terms

**FIGURE 7:**
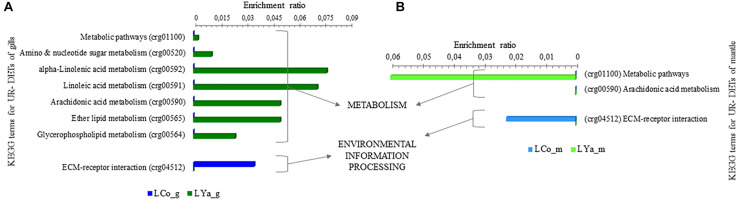
KEGG categorization of differentially expressed transcripts (DETs) for inter-location by tissue comparison. The KEGG terms represented by samples from gills tissue are in **(A)** and those represented by samples from mantle are in **(B)**. LCo, local individuals from Cochamó and LYa, locals from Yaldad. _m, mantle samples and _g, gill samples.

The inter-location by tissue comparison revealed a lower number and diversity of KEGG terms than intra- location. Thus, 1 out 8 KEGG terms were represented by 2 UR- DETs of the LCo_g samples, and 7 were represented by 16 UR- DETs of LYa_g. Contrarily, 1 out 3 KEGG terms were represented by 1 UR- DETs of LCo_m samples, and 2 represented by 4 UR- DETs of LYa_m (Supplementary Table 9). Figure 7 shows the differences, expressed as enrichment ratio (input number/background input number), between KEGG terms represented by UR- DETs, resulting from the inter- location by tissue comparison. KEGG terms represented by these samples were involved in environmental information processing through signal signaling molecule interaction and lipid and carbohydrate metabolisms. Concerning gill samples, the exclusive KEGG term represented by the samples of LCo_g was the ECM-receptor interaction (crg04512), related to signal signaling molecules interaction, involved with environmental information processing. Contrarily, KEGG terms represented by samples of LYa_g, involved with metabolism, were those related to linoleic (crg00591), alpha-linolenic (crg00592), and arachidonic acids (crg00590) (Figure 7A). Like LCo_g, samples of LCo_m also showed that their UR-DETs represented only one exclusive KEGG term, the ECM-receptor interaction (crg04512), while the UR- DETs of LYa_m represented in metabolism with only global metabolic pathways (crg01100), and arachidonic acid metabolism (crg00590) (Figure 7B).

#### KEGG Terms by Location

**FIGURE 8:**
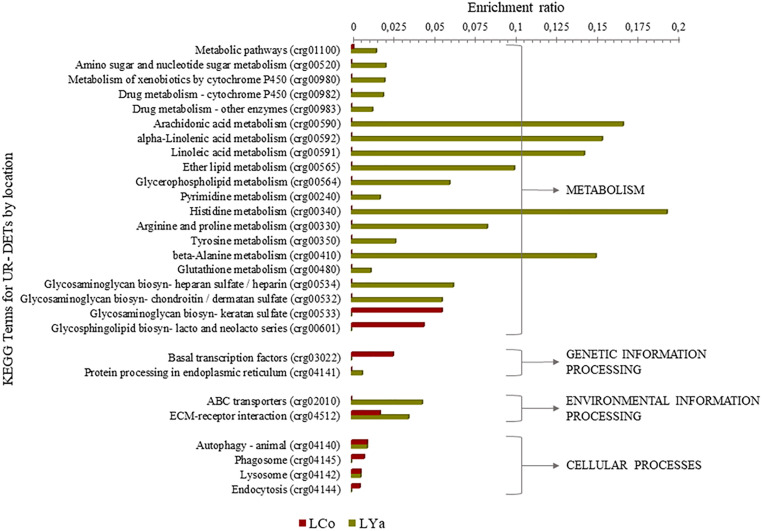
KEGG categorization of the differentially expressed transcripts (DETs) of the comparison by location. LCo, local individuals from Cochamó and LYa, locals from Yaldad.

Regardless of tissue samples, the diversity of KEGG terms between locations was higher than those intra- and inter-location by tissue comparisons. Thus, 9 out 28 KEGG terms were represented by 11 UR- DETs of Cochamó, and a higher number (23) represented by 85 UR- DETs in Yaldad (Supplementary Table 10). Figure 8 shows the differences, expressed as enrichment ratio (input number/background input number), between the KEGG terms represented by UR-DETs for this comparison. In addition to metabolism, environmental information processing and cellular processes, these samples are involved with genetic information processing. The most relevant KEGG terms represented by the samples of LCo, involved with metabolism, were those related to glycan biosynthesis through keratan sulfate (crg00533), and glycosphingolipid biosynthesis-lacto and neolacto series (crg00601). KEEG terms involved with environmental information processing were related to signaling molecules and interaction through ECM-receptor interaction (crg04512). KEGG terms involved with genetic information processing, were related to protein processing in the endoplasmic reticulum (crg04141). KEGG terms involved with cellular processes, were related to transport and catabolism through phagosome (crg04145) and endocytosis (crg04144). The diversity of KEGG terms represented by samples from LYa was higher than those from LCo, which is likely due to the number and diversity of KEEG terms mainly involved with functional metabolism category. Thus, KEGG terms related to the metabolism of carbohydrates, lipids, xenobiotics biodegradation, nucleotide, amino acid and glycan biosynthesis were exclusive of LYa. Among them, the most relevant were those involved with the metabolism of amino sugar and nucleotide sugar (crg00520), arachidonic acid (crg00590), xenobiotics (crg00980) and drugs (crg00982) by cytochrome P450, histidine (crg00340), arginine and proline (crg00330) and beta-alanine (crg00410), glycosaminoglycan biosynthesis through heparan sulfate/heparin (crg00534) and chondroitin/dermatan sulfate (crg00532). Contrary to LCo, KEGG terms involved with genetic information processing were related to transcription through basal transcription factors (crg03022), and KEGG terms involved with environmental information processing were related to membrane transport through ABC transporters (crg02010). KEGG terms, involved with cellular processes, were related to transport and catabolism through animal autophagy (crg04140), and lysosome (crg04142).

### Genetic Variants

Table 1 summarizes the characteristics of the assembled transcriptome for *Mytilus chilensis* individuals from both locations. The Cochamó transcriptome consisted of 449,180,889 trimmed raw reads assembled in 339,916 contigs, with an average of 524 bp and 178.06 Mb. For Yaldad, 441,799,205 reads assembled in 327,650 contigs, with an average of 534 bp, and a total of 175.03 Mb. Besides site-specific assemblies mapped over the reference gene library, the analysis also considered mapping over 7,900 DETs selected from the differential expression analysis.

31,186,906 trimmed raw reads mapped in Cochamó represent 6.94% of the total, whereas 34,379,475 reads mapped in Yaldad correspond to 7.79%. A total of 2,346,171 genetic variants (GVs) were detected in Cochamó, distributed as follows (Table 2A): 88.5% single nucleotide variant (SNV), 4.7% multiple nucleotide variant (MNV), 3.2% deletions, 3.2% insertions, and 0.4% of replacements. In Yaldad, 2,221,133 GVs were distributed as: 88.5% SNV, 4.7% MNV, 3.2% deletions, 3.3% insertions, and 0.4% replacements. The mapping for Cochamó totaled 2,978 GVs with frequency (f) > 0.99, whereas Yaldad exhibited 3,610.

**TABLE 2:** Genetic variant detected in assemblies of Cochamó and Yaldad, mapped over **(A)** the reference library and **(B)** selected differential expressed transcripts (DETs).

(A) Genetic variant measurement from reference library mapping.				
Variant	Cochamó		Yaldad	
		
	Count	f > 0.99	Count	f > 0.99
SNV	2,076,720	2,667	1,964,712	3,221
MNV	110,941	167	103,652	217
Deletion	74,750	65	70,538	83
Insertion	74,991	65	74,192	78
Replacement	8,769	14	8,039	11
Total	2,346,171	2,978	2,221,133	3,610
**(B) Genetic variant measurement from DETs mapping.**				
**Variant**	**Cochamó**		**Yaldad**	
		
	**Count**	**f > 0,99**	**Count**	**f > 0,99**

SNV	220,866	1,930	212,306	1,787
MNV	21,653	128	20,645	116
Deletion	5,262	18	5,111	20
Insertion	5,421	11	5,412	14
Replacement	850	4	798	3
Total	254,052	2,091	244,272	1,940

*SNV, single nucleotide variant; MNV, multiple nucleotide variants; f, frequency of detection.*

The GVs analysis for Cochamó considered over 7,900 DETs (Table 2B), showing a total of 254,052 GVs distributed as: 86.9% SNV, 8.5% MNV, 2.1% deletions, 2.1% insertions and 0.3% replacements. Overall, 2,091 GVs (0.82%) showed f > 0.99 and were distributed in 169 annotated DETs (Supplementary Table 11), 44 of them found in the transcribed sequence of Flavin-containing monooxygenase FMO GS-OX-like 9 (GSXL9, GenBank accession Q9FF12), 34 in the Heat shock 70 kDa protein 12B (HS12S, GenBank accession Q96MM6), 21 in the Cytochrome P450 10 (CP10, GenBank accession P48416), and 19 in the Heat shock 70 kDa protein 12A (HS12A, GenBank accession Q8K0U4). The GV analysis for Yaldad showed a total of 244,272 GVs, distributed as 86.9% SNV, 8.5% MNV, 2.1% deletions, 2.2% insertions and 0.3% replacements. Overall, 1,940 GVs (0.79%) showing f > 0.99 and were distributed in 150 annotated DETs, 48 of them found in the Phosphoenolpyruvate synthase regulatory protein (PSRP, GenBank accession Q0A7F4), 35 in the GTPase IMAP family member 4 (GIMA4, GenBank accession Q9NUV9) and 25 in the von Willebrand factor D EGF domain-containing protein (VWDE, GenBank accession Q8N2E2).

## Discussion

This comprehensive transcriptome analysis in the native blue mussel *Mytilus chilensis*, shows tissue-specific (gill and mantle) and location-specific gene expression. Its aquaculture exploitation depends entirely on a limited number of seed sources based on the north and south of Chiloé Island. Therefore, the transcriptome assembled represents individuals from farm-impacted seedbeds in Cochamó (LCo), at the Reloncaví fjord (northern Chiloé Island), where most seed collection centers exist, and Yaldad (LYa, southern Chiloé Island). Centers in the former area exhibit low genetic divergence, attributed to gene flow due to aquaculture practices, lower geographic distance between the seed centers, and lower environmental variability (Araneda et al., 2016). Instead, Yaldad is the oldest seedbed in southern Chiloé, where the industry began, and very likely due to the higher extractive pressure over the years, the site exhibits higher levels of endemism. Besides, artificial seed collectors compete with natural mussel beds for recruits and settlers, which would explain its reduced size (Astorga et al., 2020).

A previous study using 38 outlier SNPs under putative directional selection, obtained by RAD- Seq, suggested adaptive population divergence (F_*ST*_ = 0.155) when seeds from a center in Reloncaví fjord (Canutillar, close to Cochamó) and other close to Yaldad (Canal Coldita) where compared (Araneda et al., 2016). Our study gives practical support to the proposed adaptive divergence, providing an extensive list of candidate genes controlling multiple functional traits in each site that may shed light on how these farm-impacted seedbeds sources might be affected by translocations and climatic oscillations. Moreover, in the absence of a debugged *Mytilus chilensis* genome sequence (Murgarella et al., 2016; Li et al., 2020), the novel *M. chilensis* genomic resource identified in this study complement those available for the *M. edulis* species complex (assembled from allopatric populations), the northern hemisphere representatives of the genus (Knöbel et al., 2020). For example, the number of contigs after filtering (339,916 for LCo and 327,650 for LYa) was within the range of those reported for other species, such *M. trossulus* (437,827), *M. edulis* (353,339) and *M. galloprovincialis* (290,267). However, this study reports a higher number (189,743) of consensus contigs in the reference gene library than those reported for *M. galloprovincialis* (151,320) obtained from four different tissues (Moreira et al., 2015).

The results explain functional genomic aspects of farm-impacted *Mytilus chilensis*; such as the differentially expressed annotated genes (DETs), with multiple site-specific monomorphic genetic variants (GVs) in their transcripts. Since genetic diversity is the fuel for population adaptation and evolutionary potential, monitoring such variability at multiple functional loci should help in many ways, for example, to monitor translocations’ impact by identifying hybrid or backcrossed individuals in farm-impacted areas (Ottenburghs, 2021). Simultaneously, it should help in translocation traceability to improve exploitation practices and, eventually, to restore natural seedbeds with reduced size and impoverished genetic diversity such as Yaldad (Astorga et al., 2020). The *M. chilensis* transcriptome differentiated in Cochamó and Yaldad with a subset of 1,722 (Bonferroni corrected p_*value*_ ≤ 0.05), of which 665 (fold change ≥ | 4|), were selected as differentially expressed transcripts (DETs), representing annotated genes linked to traits involved in different biological functions. Additionally, this transcriptome evidenced that in both intra- and inter-location by tissue comparisons, the number of DETs was higher in gill samples than those from the mantle of individuals from both locations. Such differences affect metabolism, genetic and environmental information processing, and cellular processes. They are likely to be relevant in local adaptation given the north-south natural oceanographic barrier in the island (Castillo et al., 2015; Martínez et al., 2015; Lara et al., 2016), expressed primarily in temperature, salinity, water circulation (age), and concentration of chlorophyll-a; parameters that are relevant for mussel survival and reproductive performance. Studies in nature and laboratory, have evaluated *M. chilensis* response to temperature (Duarte et al., 2014; Navarro et al., 2016; Mlouka et al., 2019), salinity (Duarte et al., 2018), acidification (Castillo et al., 2017; Díaz et al., 2018; Mellado et al., 2019), and toxic substances (Núñez-Acuña et al., 2013). Diverse predators affect mussel survival (Robson et al., 2010; Curelovich et al., 2016; Riccialdelli et al., 2016) and the seasonal occurrence of different toxins due to toxic algal blooms.

Transcriptomic differences between Cochamó and Yaldad show that the expected translocation-driven genetic homogenizing effect between them is counter-balanced by the many environmental pressure listed above. Although the study did not intend to show a causal genotype-environment association, but the many candidate genes identified offer multiple opportunities to perform such a study. Along the same line of reasoning, tissue-specific transcript differences reveal complex, specialized, plastic and adaptive functions of both tissues. For example, the results showed that samples from gill tissue exhibited a higher divergent transcriptome than mantle since the large number of enriched processes found by KEGG categorization. It might be due to gills are in constant contact with the surrounding habitat and exposed to stress factors, microorganisms, xenobiotics or salinity changes. Similar results were observed for *M. galloprovincialis* (Moreira et al., 2015). Nevertheless, many of the annotated up-regulated (UR-) DETs for both tissues and locations in this study represented fewer (4 out 6) and different functional KEGG terms categories than those reported for *M. galloprovincialis*. For example, many UR- DET in this study were assigned to metabolism and environmental information processing in gills, while in the mantle to environmental information processing involving the EMC- receptor interaction. Contrarily, many transcripts were assigned for both structure and recognition of non-self-patterns in gills, while in the mantle to reproduction and shell formation in *M. galloprovincialis* (Moreira et al., 2015). These differences might be produced by the higher number of replicates sequenced for *M. chilensi*s (3 for gill and mantle tissue) in this study than those sequenced for *M. galloprovincialis* (1 for gill and 2 for mantle). Likewise, *M. galloprovincialis* mussels were obtained from a commercial shellfish farm, and before the experiments, they were acclimatized to aquarium conditions for 1 week. Instead, individuals used in this study were from farm-impacted seedbeds, and tissues samples were obtained within 4 h after individuals’ collection.

As it is said before, this study did not intend to show a causal relationship between the transcripts expressed in multiple fitness-related traits and environmental variables. However, it is possible to relate some annotated DETs detected in this study with genes reported in other studies. This study collected mussels from the so-called comfort subtidal zone (below 4 m), as defined previously (Jahnsen-Guzmán et al., 2021), with individuals from Cochamó exhibiting a higher growth rate after 91 days from the first sampling (0.015 g/day) than those Yaldad (0.004 g/day). In both comparisons between and within locations, individuals from Cochamó up-regulated the Immune-associated nucleotide-binding protein 1 (IAN13), a known GTP-binding protein participating in the regulatory mechanism of growth control and development in eukaryotes under normal and stress conditions (Liu et al., 2008), while those from Yaldad up-regulated the Geranylgeranyl pyrophosphate synthase (GGPPS), involved with the isoprenoid biosynthesis. In this context, an experimental study (Prieto et al., 2019), investigating the molecular mechanisms of growth variation in *M. galloprovincialis*, described a fast- and slow-growth transcriptomic response characterized by the differential expression of 117 genes. Fast-growing mussels up-regulated genes related to carbohydrate metabolism and citrate cycle, while slow-growth mussels up-regulated genes related to biosynthetic processes. Contrarily, this study shows that Cochamó individuals up-regulated mainly in gills transcripts related to basal transcription factors, glycosaminoglycan and glycosphingolipids. However, in gills and mantle, the ECM-receptor interaction was over-represented, demonstrating the importance for signaling molecules processes and environmental information processing in these individuals of the DET annotated for Collagen alpha-1(III) chain (CO3A1, GenBank accession P13941). On the other hand, Yaldad individuals up-regulated genes involved with protein processing in the endoplasmic reticulum and many biosynthetic processes such as carbohydrates, lipids, nucleotides and amino acids. Moreover, these individuals up-regulated transcripts annotated for ABC transporter system functioning, drug-related and xenobiotic metabolism, involving cytochrome p450 and multi-drug resistance-associated proteins, probably revealing the presence of unspecific toxic molecules, which would be absent in Cochamó. It should be noted that Cochamó individuals experiencing a slightly higher temperature than Yaldad up-regulated DETs annotated for the Heat shock 70 kDa 12A (HS12A, GenBank accession Q8K0U4), and 12B (HS12B, GenBank accession Q96MM6) proteins, known for participating in energetic metabolism and oxidative stress processes (Hamer et al., 2004). Similar observations related to water temperature were reported in *M. galloprovincialis* and *M. edulis*, and their experimental hybrids (Mlouka et al., 2019).

Gene expression differences between individuals from Cochamó and Yaldad represent their plasticity to cope with their local environments. However, the site-specific genetic variants in transcripts associated with genes with known adaptive differences may represent functional alternatives to present or future environmental shifts. The study detected 34 site-specific genetic variants distributed along the transcriptomic sequence of the Heat shock 70 kDa 12B in Cochamó individuals. Likewise, the gene for Collagen alpha-1(XII) chain, previously correlated with salinity stress in experimental studies (Lockwood and Somero, 2011), was also up-regulated in Cochamó individuals (lower water salinity than Yaldad) and exhibited 56 site-specific genetic variants (GVs), four of them with frequency (f) > 0.99 (monomorphic). Similarly, the gene encoding for Chitin synthase, previously correlated to shell biomineralization, mechanical damage, and barriers against predators (Malachowicz and Wenne, 2019), was up-regulated in Yaldad individuals, with 134 site-specific GVs, of which eight were monomorphic. Thus, many monomorphic GVs over the whole-transcriptomic correspond to up- regulated DETs in Cochamó (2,091) and Yaldad (1,940), many of them very likely correspond to differentially expressed SNPs in the DNA segregating in the overall population, but fixed in each location. Our results suggest that the search for adaptive differences in gene expression of *M. chilensis* may need to include RNA sequence variants, despite the reported discrepancies among single nucleotide variants (SNVs) detected by DNA and RNA high-throughput sequencing data, attributed to RNA editing, polyadenylation, and others (Sheng et al., 2016). Most DNA-RNA discrepancies occur in exonic non-synonymous matching, with some documented SNV in the dbSNP or RNA editing databases (Guo et al., 2017), and the translated peptides match with discordant RNA sequences that do not correspond precisely to the DNA (Li et al., 2011). Therefore, DNA sequences that show discrepancies in their correspondent RNA not only contributes to variation in gene expression but also diversifies the proteome, i.e., reinforce the role of RNA as an intermediate between the genome and proteome, and provides a more realistic view of adaptive genome response, underlying complex phenotypes often controlled by many interacting genes.

## Conclusion

1. This first *de novo* genome-wide transcriptome of *Mytilus chilensis*, assembled from two ecologically different farm-impacted seedbeds, yielded a reference library of 189,743 consensus contigs between 201 and 16,311 bases (b), with an average of 532 b, and a total of 100.91 Mb.
2. The multiple differential expression of transcripts (DETs), detected in both gills and mantle, and the monomorphic genetic variants detected in candidate adaptive genes controlling multiple fitness-related traits in both farm-impacted seedbeds, highlights the power of selective local pressures relative to translocation-driven gene flow in shaping adaptive differences in gene expression.
3. These novel genome-wide candidate adaptive genes should help monitor farm-impacted and natural seedbeds and assess their response to environmental shifts and exploitation. Additionally, to improve translocations’ traceability to conserve or restore depleted natural or exploited seedbeds.
4. These new genomic resources and their functional genetic variants contribute with tools to design an efficient management plan for this native species, conciliating the maintenance of population adaptive difference with the sustainable industry expansion in an ecosystem with multiple perturbations.

## Data Availability Statement

The datasets presented in this study can be found in online repositories. The names of the repository/repositories and accession number(s) can be found in the article/Supplementary Material.

## Author Contributions

This research is part of MY thesis who wrote the first draft in collaboration with GG. GG and CG-E provided economic support, laboratory space, and reviewed versions of the manuscript. MY and GN-A worked on the bioinformatic analysis and interpretation of data. All authors reviewed and approved the final version of the document.

## Conflict of Interest

The authors declare that the research was conducted in the absence of any commercial or financial relationships that could be construed as a potential conflict of interest.

## Footnotes

1 http://chonos.ifop.cl

2 https://odv.awi.de

## Supplementary Material

Supplementary Figure 1 Results of the RFLP assays visualized in gel of agarose 1% for both COI amplicon before **(A)** and after **(B)** the cut of *Xba*I restriction enzime, and Me15/16 before **(C)** and after **(D)** the cut of *Aci*I.

Supplementary Figure 2 Results from TapeStation 2200 (Agilent Technologies^TM^) with the R6K reagent kit. Those RNA extracts with 260/280 and 260/230 ratio >2.0 and RNA Integral Number (RIN) estimation >9 were selected for cDNA library construction.

Supplementary Table 1 Listed raw data from sequencing available in GenBank under the Bio Project accession number PRJNA630273.

Supplementary Table 2 Intra-location by tissue comparison. CLC Genomic Workbench output data.

Supplementary Table 3 Intra-location by tissue comparison. DETs annotations.

Supplementary Table 4 Inter-location by tissue comparison. CLC Genomic Workbench output data.

Supplementary Table 5 Inter-location by tissue comparison. DETs annotations.

Supplementary Table 6 Comparison by location. CLC Genomic Workbench output data.

Supplementary Table 7 Intra-location by tissue comparison. DETs annotations.

Supplementary Table 8 Intra-location by tissue comparison. KEGG terms.

Supplementary Table 9 Inter-location by tissue comparison. KEGG terms.

Supplementary Table 10 Comparison by location. KEGG terms.

Supplementary Table 11 Genetic variants by location.
