# Supplementary figures and images for: Adaptive Differences in Gene Expression in Farm-Impacted Seedbeds of the Native Blue Mussel Mytilus chilensis
Source: Front Genet. 2021 May 20;12:666539. doi: 10.3389/fgene.2021.666539 (PMC8174845; doi:10.3389/fgene.2021.666539)

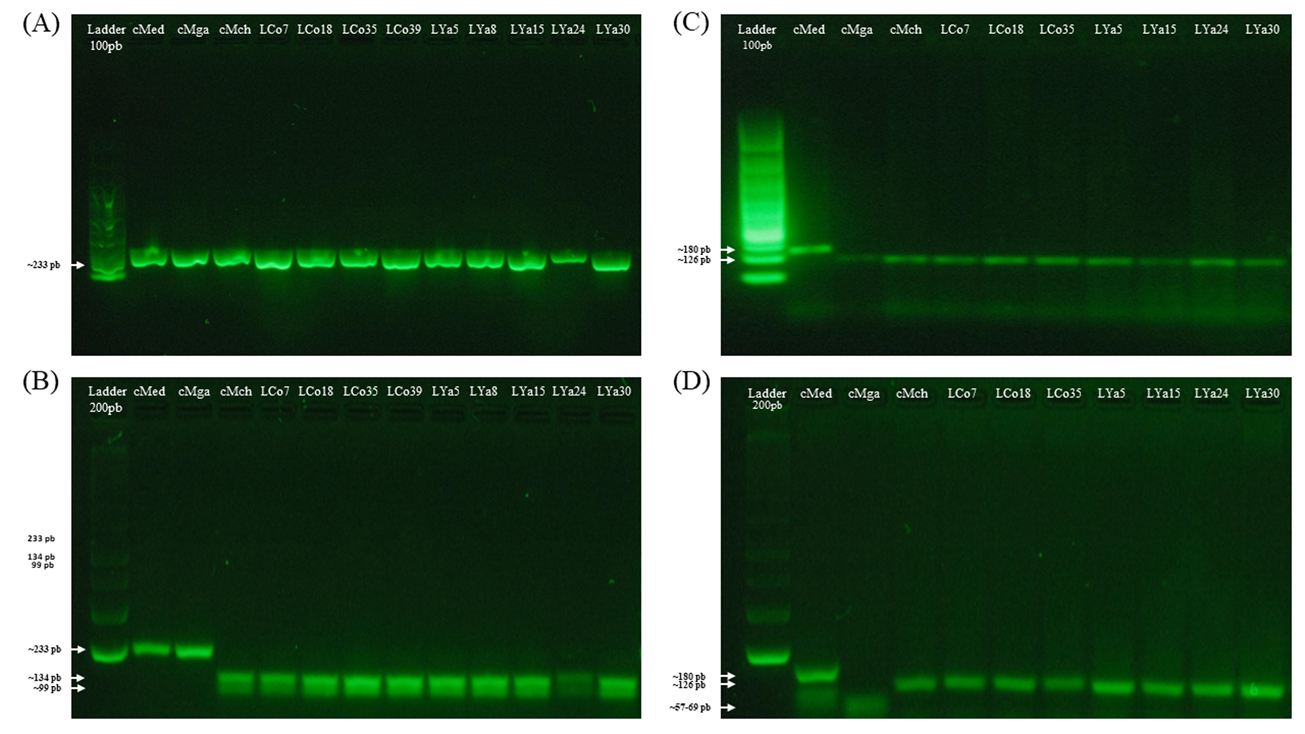

Supplement: Supplementary Figure 1 — Results of the RFLP assays visualized in gel of agarose 1% for both COI amplicon before (A) and after (B) the cut of XbaI restriction enzime, and Me15/16 before (C) and after (D) the cut of AciI. [file Image_1.TIF]

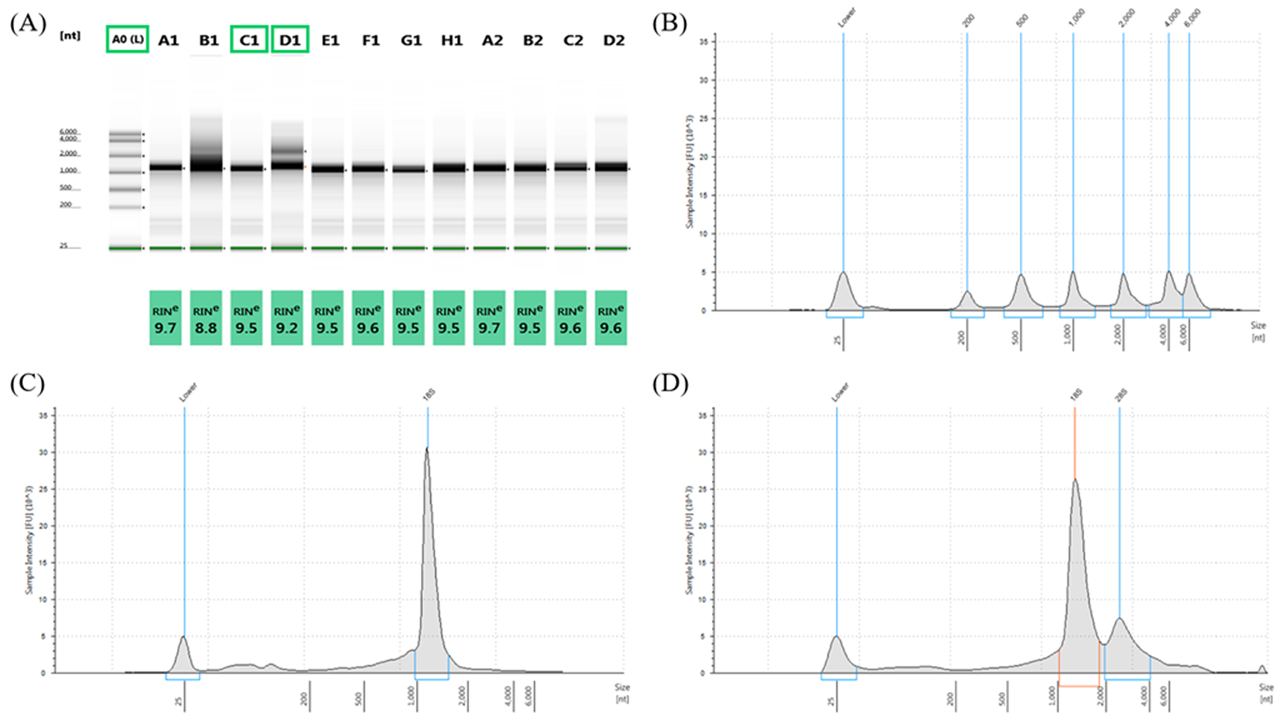

Supplement: Supplementary Figure 2 — Results from TapeStation 2200 (Agilent TechnologiesTM) with the R6K reagent kit. Those RNA extracts with 260/280 and 260/230 ratio >2.0 and RNA Integral Number (RIN) estimation >9 were selected for cDNA library construction. [file Image_2.TIF]
